# Simulation-based low-dose, high-frequency plus mobile mentoring versus traditional group-based trainings among health workers on *day of birth* care in Nigeria; a cluster randomized controlled trial

**DOI:** 10.1186/s12913-020-05450-9

**Published:** 2020-06-26

**Authors:** Emmanuel Ugwa, Mark Kabue, Emmanuel Otolorin, Gayane Yenokyan, Adetiloye Oniyire, Bright Orji, Ugo Okoli, Joseph Enne, Gabriel Alobo, Gladys Olisaekee, Adebayo Oluwatobi, Chioma Oduenyi, Adekunle Aledare, Boniface Onwe, Gbenga Ishola

**Affiliations:** 1 USAID’s Maternal and Child Survival Program/Jhpiego, Nigeria, 971 Reuben Okoya Crescent, Wuye District, Abuja, Nigeria; 2 USAID’s Maternal and Child Survival Program/Jhpiego-, 1615 Thames St, Baltimore, MD 21231 USA; 3 The Johns Hopkins Biostatistics Center, Johns Hopkins Bloomberg School of Public Health, 615 N. Wolfe Street, Baltimore, MD USA; 4 Department of Public Health, State Ministry of Health, Lokoja, Kogi State Nigeria; 5 Department of Public Health, State Ministry of Health, Abakiliki, Ebonyi State Nigeria

**Keywords:** Training, Simulation, Mentoring, Health workers, Maternal and child health, Nigeria

## Abstract

**Background:**

The aim of this study was to compare health workers knowledge and skills competencies between those trained using the onsite simulation-based, low-dose, high frequency training plus mobile mentoring (LDHF/m-mentoring) and the ones trained through traditional offsite, group-based training (TRAD) approach in Kogi and Ebonyi states, Nigeria, over a 12-month period.

**Methods:**

A prospective cluster randomized controlled trial was conducted by enrolling 299 health workers who provided healthcare to mothers and their babies on the *day of birth* in 60 health facilities in Kogi and Ebonyi states. These were randomized to either LDHF/m-mentoring (intervention, *n* = 30 facilities) or traditional group-based training (control, n = 30 facilities) control arm. They received Basic Emergency Obstetrics and Newborn Care (BEmONC) training with simulated practice using anatomic models and role-plays. The control arm was trained offsite while the intervention arm was trained onsite where they worked. Mentorship was done through telephone calls and reminder text messages. The multiple choice questions (MCQs) and objective structured clinical examinations (OSCEs) mean scores were compared; *p*-value < 0.05 was considered statistically significant. Qualitative data were also collected and content analysis was conducted.

**Results:**

The mean knowledge scores between the two arms at months 3 and 12 post-training were equally high; no statistically significant differences. Both arms showed improvements in composite scores for assessed BEmONC clinical skills from around 30% at baseline to 75% and above at end line (*p* < 0.05). Overall, the observed improvement and retention of skills was higher in intervention arm compared to the control arm at 12 months post-training, (*p* < 0.05). Some LDHF/m-mentoring approach trainees reported that mentors’ support improved their acquisition and maintenance of knowledge and skills, which may have led to reductions in maternal and newborn deaths in their facilities.

**Conclusion:**

The LDHF/m-mentoring intervention is more effective than TRAD approach in improving health workers’ skills acquisition and retention. Health care managers should have the option to select the LDHF/m-mentoring learning approach, depending on their country’s priorities or context, as it ensures health workers remain in their place of work during training events thus less disruption to service delivery.

**Trial registration:**

The trial was retrospectively registered on August 24, 2017 at ClinicalTrials.Gov: NCT03269240.

## Background

As there is limited number and quantity of skilled birth attendants in Nigeria, an evidence-based approach is needed to train health workers to improve competencies and maternal/newborn outcomes [1, 2]. Doctors, nurses and community health extension workers (CHEWs) are commonly trained on *day of birth* care. Considering the high maternal and newborn mortality and morbidity figures in Nigeria, there is need to train birth attendants on high-impact interventions using competency-based approaches to improve the quality of maternal and newborn care and reduce maternal and perinatal/neonatal mortality [3–6]. It is expected that all health workers should be trained to the necessary level of competence to perform life-saving procedures on Basic Emergency Obstetric and Newborn Care (BEmONC) functions. These include newborn resuscitation and management of maternal bleeding after birth.

Despite continuous training of health workers, maternal and newborn health outcomes have remained suboptimal [7]. Health workers in Nigeria are usually trained using traditional lecture-based, offsite approach where limited number of health workers is trained from each facility at a time and the quality of care may not be improved to the desired level though the use of this approach [3]. Apart from the high cost of the offsite training approach [7–10], health workers’ absenteeism from work to attend offsite training remains a concern for health facility managers [9, 10]. In addition, health workers also find it difficult to practice new or updated skills because their colleagues at the facility have not received the same training on the relevant skills and approaches. In addition, step-down training by the few trained providers most often does not happen as intended due to lack of will, lack of time and management support among other factors. Moreover, health worker turnover, attrition and transfers also affects the quality of services particularly, when providers who have been trained are transferred out of the facility or when they leave by themselves.

Similar studies conducted is Ghana and Uganda (7,8) showed promising results with the onsite simulation-based low-dose, high-frequency plus mobile mentoring training (LDHF/m-mentoring) approach. In these studies health workers’ competencies were better with the LDHF/m-mentoring approach compared with the TRAD approach for skills assessed such as newborn resuscitation and postpartum haemorrhage (PPH). The present study assessed additional skills such as active management of labour, management of eclampsia, and utilizes a more rigorous methodology in order to improve the strength of the evidence. A qualitative component is also included to triangulate the findings. There is limited evidence from developing countries including Nigeria on facilitators, barriers, and effectiveness of simulation-based LDHF/m-mentoring learning approaches in improving maternal and newborn health, including *day of birth* care*.* In the few studies where the LDHF/m-mentoring approach has been tested, sample sizes have been limited and methodological approaches have not been standardized [7]. The studies should be adequately powered and use rigorous designs to answer the research questions [7].

The LDHF/m-mentoring approach used in this study is described in the study protocol manuscript. This approach primarily involves simulation education, onsite, more interactive, and team-oriented training [11]. Simulation based medical education is an educational activity that utilizes simulation aids such as human anatomical models or manikins to replicate clinical case scenarios [12]. Simulations create opportunity for participants to improve competence through deliberate practice and serve as alternative to real case scenario especially for rarely practiced skills. In this instance, trainees may make mistakes and learn from them without the fear of injuries or harm to the patient [12].

## Methods

### Study objectives

The aim was to compare the knowledge and skills competencies of health workers in maternal and newborn care on *day of birth* after the LDHF/m-mentoring versus the TRAD training approaches in Ebonyi and Kogi states, Nigeria. The primary outcomes were increase in knowledge, clinical skills, and retention of clinical competency at 3- and 12-months post-training. The secondary outcomes measured were facilitators of and barriers to the LDHF/m-mentoring training approach at individual and institutional levels.

Our hypothesis was that LDHF/m-mentoring (Group 1: intervention arm) results in better knowledge and skills outcomes compared to the TRAD approach (Group 2: control group).

### Study setting

The study was conducted in Ebonyi and Kogi States, in Nigeria. Ebonyi State has a population of 2.8 million and is in the South-East zone. Kogi state has a population of 4.3 million and is in the North-Central zone. At the time of the study, the Maternal and Child Survival Program (MCSP), in partnership with the Federal Ministry of Health, Ebonyi and Kogi States Ministries of Health and Professional Associations supported 120 health facilities across the two states, of which 60 were selected to be part of the study, 30 in each state. The MCSP was a global, $560 million, 5-year cooperative agreement funded by the United States Agency for International Development (USAID) to introduce and support scale-up of high-impact health interventions among USAID’s 25 maternal and child health priority countries, as well as other countries.

### Study design

This was a prospective cluster randomized controlled trial. It isa mixed- method study with quantitative and qualitative data collection approaches utilized. The study was conducted between October 2016 and November 2017 in sixty health facilities that were randomly selected and assigned to either the intervention arm or the control arm. A brief description of the methodology, the training approaches, eligibility criteria and randomization are provided; these are described extensively in the published study protocol [11].

### Training approach 1 and data collection: simulation based LDHF/m-mentoring training of participants – group 1 or intervention group

As described by the authors in a previous study [11], the principles of the LDHF/m-mentoring approach include:

1. Competency-focused learning activities concentrates on what providers “need to know”—eliminating what is “nice to know.”
2. Simulation- and case-based learning focuses on skills practice, problem-solving, role-play, and other interactive exercises. Dosing and frequency depend on topic, extent of the learning gaps, and learner characteristics.
3. Appropriately spaced, brief periods of learning deliver targeted information in 1 day or over several days.
4. Team-focused training ensures that all providers have updated clinical practice and can work together to implement improvements in care.
5. Facility-based training decreases absenteeism, improves teamwork, addresses onsite barriers, and promotes changes to provider performance.
6. Ongoing practice and quality improvement activities reinforce learning and transfer to clinical practice.
7. Facility-based peer staff, coaches others as they practice or engage in interactive exercises after learning to increase compliance and improve performance and outcomes.

#### Consent

Oral consent was obtained from the study participants prior to training and assessment (measurement).

#### Training for group 1 LDHF/m-mentoring arm

The training for group 1 LDHF/m-mentoring arm was conducted for the entire team of health workers rendering maternal and newborn care at the health facility, but only those who met the study inclusion criteria were assessed. The training was divided into two “low-dose” training courses of four days each, with additional time for assessment as needed and was conducted at the health facilities using the BEmONC package. The BEmONC package include training on (i) administering parenteral antibiotics (ii) administering uterotonic drugs for active management of the third stage of labour and prevention of postpartum haemorrhage (iii) use of parenteral anticonvulsants for the management of pre-eclampsia/eclampsia.

(iv) Manual removal of placenta (v) removal of retained products (e.g. manual vacuum extraction, dilatation, and curettage), (vi) performing assisted vaginal delivery and (vii) performing basic neonatal resuscitation [13].

The LDHF/m-mentoring approach uses in-service learning updates to deliver information based on local needs during short, structured, onsite, interactive learning activities that involve the entire team and are spaced over time to optimize learning. It also involves brief, ongoing activities (e.g., skills practice, team drills, games, and quality improvement activities) at the work place to sustain learning and support clinical decision-making [11].

**Fig. 1:**
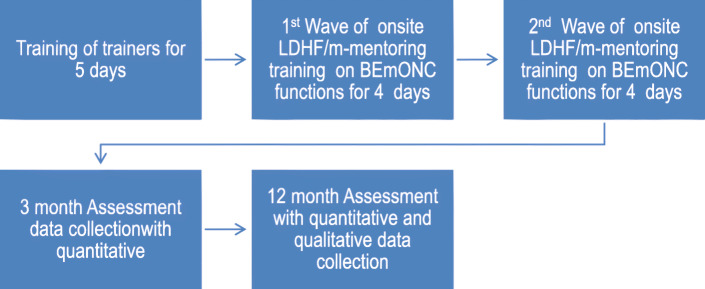
Training approach 1 and data collection: Simulation based LDHF/m-mentoring training of participants – group 1 or intervention group (n1 = 172)

The training was conducted for an initial four days with emphasis on normal uncomplicated cases. After 1 month this was repeated for another four days to emphasize more complicated skills (Fig. 1). The training techniques were modified to shift the emphasis from knowledge to practice. This was a competency-based training approach that has been used in Jhpiego’s (affiliate of Johns Hopkins University) training with the aim of improving and retaining the skills of the health worker. The trainer practices the skills while the trainee observes. The trainee then practices using the anatomical models (manikins) while the trainer observes. At the end, the trainer debriefs and provides feedback to the trainee.

The simulation-based clinical practice creates opportunity to improve competence through deliberate practice on anatomical models used to mimic real clinical scenario. The trainee repeats the practice until competency is achieved. The Peer Practice Coordinators (PPCs) received technical update in LDHF/m-mentoring including the use of session plans, case scenario and anatomical models (MamaNatalie/NeoNatalie) models to conduct simulation practices. The time spent on lectures was reduced and time spent on hands-on practice was increased. During the one-month interval between training courses, health care workers had opportunity to practice what they learned through high-frequency simulation-based peer practice and reinforce their competencies through high-frequency simulation-based practices of 2–3 times weekly. The PPCs completed the practice log.

#### Mobile mentoring

The approach also uses mobile phone mentorship calls and reminder text messages to the trainees to reinforce gains made from training and resolve emerging issues at the workplaces. All the trained health workers in the LDHF/m-mentoring arm participated in mobile Mentoring (m-mentoring), which consisted of receiving weekly reminder messages and quiz questions on the topics reviewed via SMS messaging. Also the PPCs received structured, monthly half-hour mentoring calls from a trainer/master mentor that provided remote support, answering questions, providing guidance and reinforcing key messages.

#### Measurement

The participants undertook a pre-training assessment consisting of multiple choice questions (MCQs) and objective structured clinical examinations (OSCEs) to assess their baseline knowledge and skills on BEmONC. The MCQ contained a set of multiple choice questions to test trainees’ knowledge. The OSCEs involved the use of checklists to evaluate trainees’ demonstration of clinical skills to ensure that each step is correctly and completely carried out. These tools were adapted from previous studies and validated by the researchers and trainers [6, 7, 12]. The participants were assessed at three different times after training by clinicians whose clinical skills have been standardized (Fig. 1). The skills and knowledge of the participants were assessed immediately after training and at three and 12 months using MCQs and OSCEs. The assessments tested their knowledge and skills on conduct of normal delivery, active management of the third stage of labor (AMTSL), neonatal resuscitation, case management of pre-eclampsia and eclampsia (PEE) and management of PPH (e.g. manual removal of placenta, internal bimanual uterine compression and compression of the abdominal aorta). The questions answered correctly and procedure done competently was scored over a total of 100%. A test score of ≥80% was accepted as level of competence. The pre-training and immediate post-training assessments results were compared. Trainees’ satisfaction with the simulation-based LDHF/m-mentoring training approach was determined using satisfaction (quantitative) survey. The scores at each assessment were collected using validated tools and recorded in real-time on android devices and sent to a central server after verification.

#### Qualitative data

Qualitative data were collected through six focus group discussions (FGD) comprising 8–10 participants per group purposively selected from LDHF + m-mentoring arm at 12 months. The participants were invited via telephone and provided with the study information including its aims and procedures. All the participants gave consent. The FGDs were used to collect data on experiences and satisfaction of trainees with the LDHF/m-mentoring training approach. The enquiry was based on the high-frequency practice sessions with simulators, mobile mentoring through short messages and quizzes, overall impressions of the LDHF/m-mentoring approach, and how it could be improved. In-depth interviews (IDIs) were conducted with PPCs to collect data on their experience managing simulator practice sessions, interactions with trainers/master mentors, m-mentoring, changes made in clinical practice after the training, success, challenges and their overall impression about LDHF/m-mentoring training approach. With regard to trainers, data were collected about their experiences with the LDHF/m-mentoring training approach, successes and challenges with mobile mentoring to support the PPCs, and the effectiveness of approach in building the capacity of the health workers. The data were collected at the participants’ workplaces. Only the interviewer and the respondents were present at a private place where the interviews were conducted. Interview guides were adapted from previous work and pretested. Repeated interviews were not carried out. Audio-recorders were used during interviews to help during recall. Each interview lasted for 45-60 min. The qualitative data was collected until data saturation was reached. The interviews were transcribed and data analyzed. The Transcripts were not returned to participants for comments.

### Training approach 2 and data collection: traditional group training of participants (TRAD arm) – group 2 or control group

#### Consent

Oral consent was obtained from the study participants prior to training and assessment (measurement).

#### Training of group 2 (TRAD) arm

**Fig. 2:**
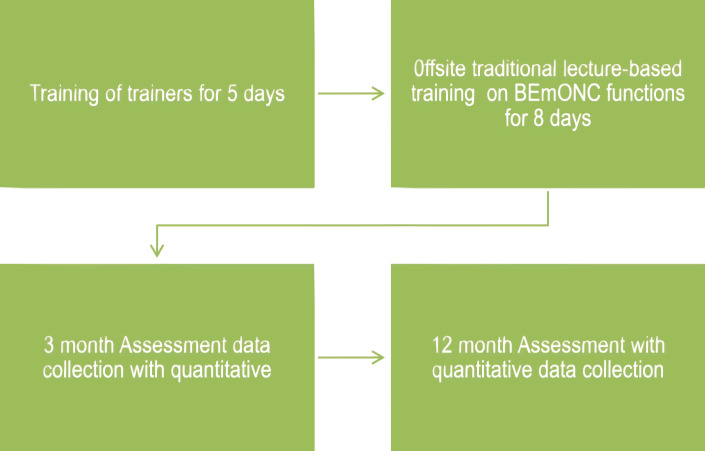
Training approach 2 and data collection: Traditional off-site training of participants – group 2 or control group (n2 = 127)

The traditional training approach consisted of eight straight days of lectures with practice sessions on simulators, outside the participants’ workplace, usually in a hotel (Fig. 2). The group had lectures and practice sessions on conduct of normal delivery, AMTSL, neonatal resuscitation, case management of PEE and management of PPH. The BEmONC package and clinical observation checklists were used during the training sessions as was done for the intervention arm. Emphasis was not made on practice sessions at the health facilities when they returned. They did not participate in m-Mentoring.

#### Measurement

Participants undertook a pre-training assessment consisting of MCQs and OSCEs to assess their baseline knowledge and skills on BEmONC. The MCQs and OSCEs were similar to those administered to the intervention group. At the end of the eight-day training, the participants had an immediate post-training assessment which included MCQs and OSCEs. As described for the intervention arm, the questions answered correctly and procedure done competently were scored out of a total of 100%. These were recorded in real-time on android devices and sent to a central server after verification. The assessments results were compared. Trainees’ satisfaction survey was also conducted. The participants were then assessed at different points after training. The participants were also assessed at three different times after training by clinicians whose clinical skills have been standardized.

The skills and knowledge of the participants were assessed immediately after training and at three and 12 months using MCQs and OSCEs. The assessments tested their knowledge and skills on conduct of normal delivery, AMTSL, neonatal resuscitation, case management of PEE and management of PPH (e.g. manual removal of placenta, internal bimanual uterine compression and compression of the abdominal aorta). The questions answered correctly and procedure done competently was scored over a total of 100%. A test score of ≥80% was accepted as level of competence. The pre-training and immediate post-training assessments results were compared.

Participants in both study arms were trained and assessed by senior clinicians (mainly obstetricians, pediatricians and midwives) whose knowledge and skills were standardized. The assessment tools were pre-tested among 25 health workers from health facilities that were not part of the study. The assessment checklists for clinical skills were modified from the versions used in Ghana LDHF study [7] and piloted in accordance with the Nigeria context by the research team and data collectors. At the time of the training, MCSP did not support any interventions in these facilities that are likely to cause contamination. Other quality improvement interventions only happened at the non-study facilities at the time of the study.

The assessors were blinded to the groups the participants were assigned to as they conducted the assessments.

### Eligibility criteria

The sixty health facilities were selected from a sampling frame of the 120 MCSP-supported health facilities in the two states. These health facilities represented all three levels of the healthcare system in Nigeria (Primary health care or PHC, secondary, tertiary). The health workers were drawn from among those working in labor and delivery sections of the participating health facilities in the two states. In addition, the health workers had spent at least six months in the health facility providing maternal and newborn care.

### Randomization

The unit of randomization in this study was the health facility. The 60 facilities were matched based on locations and level in the health care system, then divided into nine strata taking into consideration the three geopolitical wards within the states. Thereafter, each stratum was randomized to either intervention or control arm using randomly permuted blocks in a ratio of 1:1 so as to achieve balance in geographical location and types of health facilities in the two study arms. Those assessing outcomes were blinded to the training methodology used for the health facilities. Since there were at times less than three skilled birth attendants in some health facilities, all health workers employed in the maternity or newborn units in such facilities who met the inclusion criteria were selected from the randomized health care facilities [11].

### Sample size

The details on the computation of the sample size, including the assumptions are provided in the study protocol manuscript [11]. Briefly, the required number of participants was computed as the average number of health workers to be included from each of the 60 facilities (30 per arm) using a test of two proportions. The percent of competent health workers was estimated at 50% in the control group. Study power was set at 80% to reject a null hypothesis that the proportions of competent health workers are equal in the two study arms against the alternative hypothesis of 20 percentage point difference in proportions of competent health workers between the two study arms - effect size. The significance level was set at 0.05. Sample size computation was done using PASS statistical software. The correlation of health workers competency in each health facility was assumed to be 0.05 given that some facility-level factors are shared by the health workers working together might influence how they perform certain tasks. We assumed there are about four health workers selected per facility, thus 240 health workers sampled across the two groups. An adjusted sample size of 300 participants (150 per study arm) was arrived at after factoring in potential 20% drop-out during follow up period after baseline. More health workers were recruited from Kogi State since it had a larger population than Ebonyi. Study participants recruited from each facility included the maternity unit head, wherever possible, and two others to ensure that the other team members received the necessary support to practice.

### Data analysis

For both arms, composite scores were computed for the infection prevention and the BEmONC functions, namely; skills in conducting normal delivery, AMTSL, neonatal resuscitation, case management of PEE with magnesium sulphate (MgSO4), and the management of primary PPH Percent scores were computed per participant based on either questions answered correctly or procedures done competently depending on the assessment tool used.

To describe categorical variables, counts and simple proportions were used. Mean and standard deviations as well as median and interquartile range were used to summarize data on continuous variables. Percentage of those achieving the required competency level of ≥80% post-training scores in MCQs and OSCEs were computed and compared across the two study arms. A generalized linear model was used to test the differences between arms on MCQ and OSCE scores at 12 months using a group indicator as the main predictor in the model. Furthermore, adjustments were made to account for facility-level and health worker characteristics that might have influenced the two study arms at baseline and could be strongly correlated with the outcome scores. A significance level of < 0.05 was set for statistical significance. A longitudinal model that accounts for intra-provider correlations over time as well as within-facility correlations was developed. This model assessed the change in scores over time post-training. The model is appropriate, since it estimated using generalized estimating equations with working exchangeable correlation structure.

Regarding qualitative data, the translated Microsoft Word versions of the transcripts were imported into ATLAS.ti software (version 8.0) for content analysis. The codebook was developed by two (2) qualitative researchers, the data analyst and a second coder, using a priori codes developed from the research aims and questions, and the interview guides. In order to enhance validity, the researchers adopted a 1st tier triangulation (of researchers) and ensured a well-documented audit trail of materials and processes. To ensure reliability, constant data comparison and comprehensive data use were done. Reliability checks were performed by each coder independently and by re-coding documents already coded by the other.

## Results

**Fig. 3:**
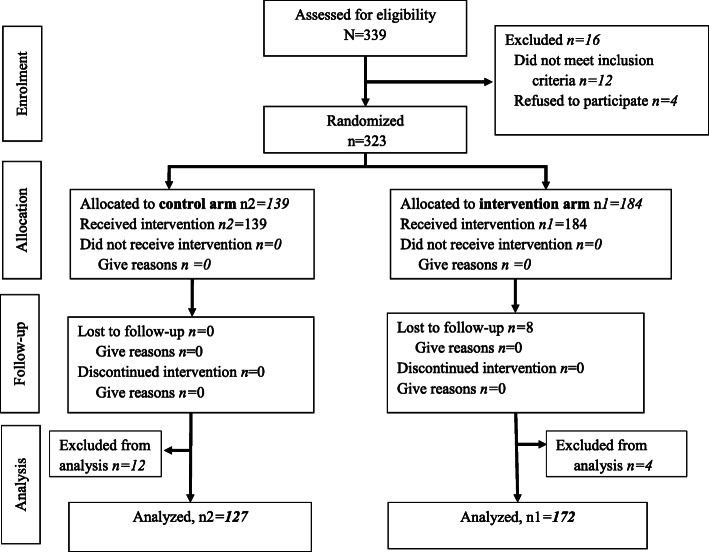
Consort flowchart of enrolment of study participants and data analysis

Two hundred and ninety-nine (299) participants completed the study out of the original 323 providers who were randomized to different study arms; LDHF/m-mentoring arm = 172; TRAD arm = 127 (Table 1 and Fig. 3). Both arms had similar socio-demographic characteristics Table 1).

**Table 1:** Baseline characteristics of study sample of providers by study arm, *N* = 299

**Characteristic of Health Workers**	**LDHF/m-mentoring (n1 = 172)** **n (%)**	**TRAD (n2 = 127)** **n (%)**	***p*value**
**State or Location**			
Ebonyi	63 (36.6)	33 (26.011)	*0.05*^a^
Kogi	109 (63.4)	94 (74.0)	
**Type of Facility**			
Primary health center	66 (38.4)	60 (47.2)	
General/Mission hospital	90 (52.3)	49 (38.6)	
Tertiary hospital	16 (9.3)	18 (14.2)	*0.05*^a^
**Mean (SD) Age: years**	41.0 (10.3)	40.6 (8.8)	0.78^b^
**Sex**			
Male	27 (15.7)	28 (22.0)	0.16^a^
Female	145 (84.3)	99 (78.0)	
**Marital Status**			
Married	145 (84.3)	113 (89.0)	0.60^c^
Single	18 (10.4)	11 (8.6)	
Divorced	1 (0.6)	0 (0.0)	
Widowed	8 (4.7)	3 (2.4)	
**Religion**			
Christian	137 (79.7)	95 (74.8)	0.33^a^
Islam	32 (18.6)	29 (22.8)	
Other	0 (0.0)	1 (0.8)	
Missing	3 (1.7)	2 (1.6)	
Duration Since Graduation: years	12.0 (6.0–23.0)	14.0 (8.0–21.0)	0.54^d^
**Cadre/Job Title**			
Community health extension worker	59 (34.3)	55 (43.3)	0.03^*c^
Doctor	6 (3.5)	11 (8.7)	
Nurse	98 (57.0)	59 (46.4)	
Other	9 (5.2)	2 (1.6)	
Median (IQR) Duration Working at Facility since Employment: years	6.0 (3.0–10.0)	6.0 (3.0–10.0)	0.84^d^
Median (IQR) of travel time to training (minutes)^e^	60.0 (60.0–90.0)	60.0 (60.0–120.0)	< 0.001^*d^
Duration at current position: median (IQR) in years	8.0 (4.0–18.0)	9.0 (4.0–15.0)	0.76^d^

Types of tests: ^a^ = Pearson’s chi-squared; ^b^ = Two sample t test; ^c^ = Fisher’s exact; ^d^ = Wilcoxon rank-sum. SD, standard deviation; IQR, interquartile range. *,= statistical significance ^e^ = TRAD participants residing within 5 km of the training sites were not accommodated in a hotel; they travelled daily to the training site

Table 2 shows that the TRAD arm had better knowledge test scores compared to the LDHF/m-mentoring arm at baseline and immediately after the training intervention in some thematic areas, such as AMTSL and neonatal resuscitation (*p* < 0.05). However, at 3 and 12 months after training assessment, both arms were equal in knowledge acquisition and retention; no statistically significant differences were evident.

**Table 2:** Comparison of levels of knowledge between study arms across four assessment periods, *N* = 299

**Thematic Area #**	**Thematic Area**	**Total number of items**	**Assessment 1—Baseline**					
					**Unadjusted analysis**		**Adjusted analysis**	
			**Mean # correct, LDHF/m-mentoring**	**Mean # correct, TRAD**	**IRR (95%CI)**	***p value***	**IRR (95%CI)**	***p*value**
**1**	**Infection prevention**	6	3.17	2.67	1.19 (1.07, 1.32)	0.001^*^	1.17 (1.06, 1.30)	0.002^*^
**2**	**Normal birth**	8	4.17	4.01	1.04 (0.96, 1.13)	0.349	1.03 (0.95, 1.12)	0.498
**3**	**AMTSL**	4	1.79	1.73	1.03 (0.88, 1.21)	0.682	1.02 (0.88, 1.18)	0.822
**4**	**Management of eclampsia**	2	1.13	1.11	1.02 (0.89, 1.18)	0.754	1.02 (0.88, 1.17)	0.805
**5**	**Essential newborn care**	2	0.94	1.03	.92 (0.77, 1.09)	0.318	0.91 (0.76, 1.08)	0.270
**6**	**Neonatal resuscitation**	20	8.55	11.15	0.77 (0.70, 0.84)	< 0.001^*^	0.75 (0.68, 0.82)	< 0.001^*^
	**Overall score**	42	17.62	19.71	0.89 (0.83, 0.96)	0.002^*^	0.88 (0.82, 0.94)	< 0.001^*^
**Thematic Area #**		**Total number of items**	**Assessment 2—Immediate post-training**					
					**Unadjusted analysis**		**Adjusted analysis**	
	**Thematic Area**		**Mean # correct, LDHF/m-mentoring**	**Mean # correct, TRAD**	**IRR (95%CI)**	***p value***	**IRR (95%CI)**	***p value***
**1**	**Infection prevention**	6	5.01	5.04	1.00 (0.94, 1.06)	0.888	0.98 (0.92, 1.04)	0.470
**2**	**Normal birth**	8	6.51	6.76	0.96 (0.92, 1.01)	0.134	0.95 (0.90, 1.00)	0.040^*^
**3**	**AMTSL**	4	3.2	3.49	.92 (0.86, 0.98)	0.009^*^	0.90 (0.84, 0.96)	0.002^*^
**4**	**Management of eclampsia**	2	1.72	1.8	0.95 (0.89, 1.02)	0.138	0.95 (0.89, 1.01)	0.116
**5**	**Essential newborn care**	2	1.34	1.65	0.81 (0.74, 0.89)	< 0.001^*^	0.80 (0.72, 0.88)	< 0.001^*^
**6**	**Neonatal resuscitation**	20	11.29	16.21	0.7 (0.63, 0.77)	< 0.001^*^	0.68 (0.61, 0.75)	< 0.001^*^
	**Overall score**	42	24.21	30.57	0.79 (0.75, 0.84)	< 0.001^*^	0.77 (0.73, 0.82)	< 0.001^*^
**Thematic Area #**		**Total number of items**	**Assessment 3—3 months post-training**					
					**Unadjusted analysis**		**Adjusted analysis**	
	**Thematic Area**		**Mean # correct, LDHF/m-mentoring**	**Mean # correct, TRAD**	**IRR (95%CI)**	***p value***	**IRR (95%CI)**	***p value***
**1**	**Infection prevention**	6	4.54	4.24	1.07 (0.99, 1.16)	0.086	1.04 (0.96, 1.12)	0.348
**2**	**Normal Birth**	8	5.9	5.7	1.04 (0.96, 1.11)	0.342	1.01 (0.94, 1.08)	0.760
**3**	**AMTSL**	4	3.07	3.04	1.01 (0.92, 1.10)	0.853	0.97 (0.89, 1.06)	0.569
**4**	**Management of Eclampsia**	2	1.69	1.7	1.00 (0.92, 1.08)	0.932	0.99 (0.91, 1.07)	0.741
**5**	**Essential Newborn Care**	2	1.55	1.52	1.02 (0.91, 1.14)	0.781	1.00 (0.89, 1.12)	0.964
**6**	**Neonatal Resuscitation**	20	14.45	13.98	1.03 (0.96, 1.11)	0.372	1.00 (0.93, 1.08)	0.978
	**Overall score**	42	27.41	26.37	1.04 (0.98, 1.10)	0.200	1.01 (0.95, 1.06)	0.796
**Thematic Area #**		**Total number of items**	**Assessment 4—12 months post-training**					
					**Unadjusted analysis**		**Adjusted analysis**	
	**Thematic Area**		**Mean # correct, LDHF/m-mentoring**	**Mean # correct, TRAD**	**IRR (95%CI)**	***p value***	**IRR (95%CI)**	***p value***
**1**	**Infection prevention**	6	4.15	3.99	1.04 (0.95, 1.14)	0.380	1.01 (0.93, 1.10)	0.795
**2**	**Normal birth**	8	5.59	5.32	1.05 (0.97, 1.14)	0.213	1.03 (.95, 1.11)	0.470
**3**	**AMTSL**	4	2.88	2.89	1.00 (0.90, 1.10)	0.944	0.96 (0.88, 1.06)	0.426
**4**	**Management of eclampsia**	2	1.61	1.59	1.02 (0.93, 1.11)	0.730	1.00 (0.92, 1.10)	0.920
**5**	**Essential newborn care**	2	1.51	1.46	1.04 (0.93, 1.16)	0.544	1.01 (0.90, 1.13)	0.867
**6**	**Neonatal resuscitation**	20	13.83	14.02	0.99 (0.94, 1.04)	0.588	0.95 (0.91, 1.00)	0.036^*^
	Overall score	42	26.47	26.16	1.01 (0.96, 1.06)	0.642	0.98 (0.94, 1.02)	0.340

*indicates statistical significance or *p*<0.05

*IRR* incidence rate ratio, *CI* Confidence interval

**Fig. 4:**
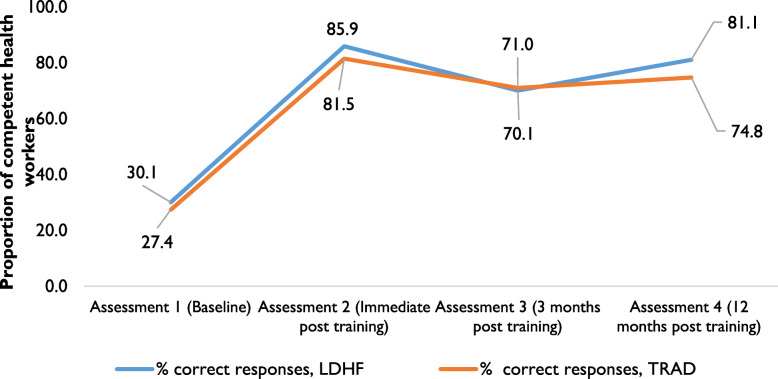
Trends in BEmONC skills mean composite scores by study arm over 12-month period (arm 1, n1 = 172; arm 2, n2 = 127)

Health workers in both arms showed improvement in overall pass rates in clinical skills competency, improving from around 30% at baseline to 75% and above at endline; difference-in-differences were statistically significant (*p* < 0.05); TRAD (from 27.4 to 74.8%) and LDHF/m-mentoring (from 30.1 to 81.1%). Overall, the observed improvement and retention of BEmONC skills was higher in LDHF/m-mentoring study arm participants (81.1%) compared to the TRAD arm participants (74.8%) at 12 months post-training (*p* < 0.05). There was a slight drop in skills competency in both arms at the three-month assessment (Fig. 4).

**Fig. 5:**
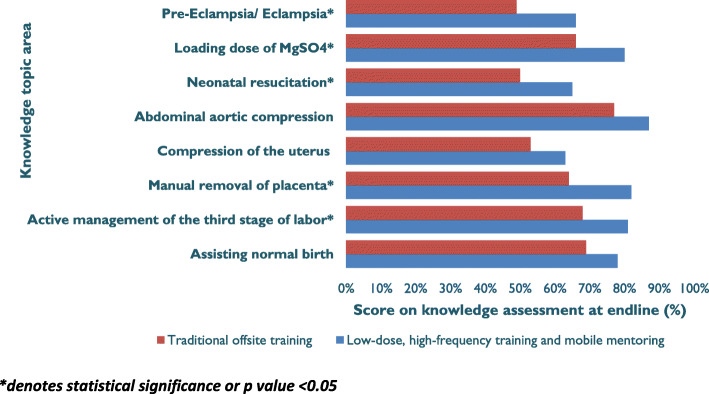
Assessment of providers’ BEmONC skills at 12 months post-training (arm 1, n1 = 172; arm 2, n2 = 127)

Figure 5 shows that overall for BEmONC skills, the LDHF/m-mentoring arms had better post-training assessment scores at 12 months post-training for assisting normal birth (including care of the newborn), AMTSL, manual removal of placenta, bimanual compression of the uterus, abdominal aortic compression, PEE management (*p* < 0.05).

Analysis codes were grouped into four thematic categories, guided by both the specific objectives, research questions and those that emerged from the data.

The results of the qualitative study are presented in four sections. The first section addressed knowledge and skill learning outcomes of birth attendants following simulation-based LDHF/m-mentoring, the second section addressed trainees’ satisfaction with successful outcomes following simulation-based LDHF/m-mentoring approach, third section addressed facilitators of LDHF/m-Mentoring approach and the fourth section addressed the barriers to LDHF/m-mentoring approach.

Excerpts from respondents are presented in italics. Each quote in the text is labeled by type of interview and State.

### Theme 1: Knowledge and skill learning outcomes of birth attendants

In both states, all the FDG participants opined that the LDHF/m-mentoring training approach enabled them gain improvement in skills/knowledge and quality of care.

An FGD participant in Ebonyi said:

*‘The training was different to me by emphasizing that the partograph is a medico-legal document.*

*The training endeared me to the use of partograph to monitor labour and the importance of documentation. We have started using partograph. Before now, we were not using partograph to manage labour’ (FGD, Ebonyi).*

### Theme 2: Trainees’ satisfaction with successful outcomes following the simulation-based LDHF/m-mentoring approach

The respondents reported reduction in maternal and neonatal morbidity and mortality as common theme across states.

*We have saved babies that would have died. Now babies can be resuscitated so more babies are surviving and we are having more successful deliveries.*

### Theme 3: Facilitators of LDHF/m-mentoring approach

Respondents identified facilitators of LDHF/m-Mentoring approach as support provided by the implementing partners through availability of equipment/supplies, use of telephone for communication, expert knowledge/skill and support from master mentors, incentives/welfare such as transport reimbursement and meals during training, support from management of facilities such as arrangement of duty rosters to enable trainees attend practices sessions and assessments through text messages and quizzes.

A respondent in Ebonyi state said:

*‘Our implementing partners are the biggest supporter of this program, they trained us.’*

### Theme 4: barriers to LDHF/m-mentoring approach

The barriers mentioned included that different work schedules prevented some trainees from attending some practice sessions and unavailability of equipment hindered some from translating what they learnt into practice.

*‘I find it difficult bringing everybody together at the same time because we run different shifts. It was very difficult to get members of the group to come together and practice due to our work schedules’ (IDI, Ebonyi).*

## Discussion

In this cluster randomized control trial, the knowledge assessment scores increased from baseline to end-line in both the study arms. The acquisition and retention of BEmONC skills differed at 12 months with the LDHF/m-mentoring arm demonstrating better performance than the TRAD arm. This study contributes to the much needed evidence base for LDHF/m-mentoring training approach in Low -and Middle- Income Countries (LMICs), adding on the finding of related studies done in Ghana [7] and Uganda [8]. As part of the intervention package, LDHF/m-mentoring study arm participants received weekly SMS reminders and quizzes along with continuous skills practice on anatomical models, which helped, reinforce knowledge on specific clinical areas over time. In contrast, the TRAD arm participants, which represented the *status-quo,* did not have the extra support, which might explain the differences in skills performance at 12 months. Furthermore, information gathered qualitatively suggests that a key benefit of m-mentoring is the ability of trainees to call their mentors at the point of care to address complications such as placenta praevia or birth asphyxia. This close mentor-mentee relationship might also have helped the mentees to develop confidence in these clinical skills, which demonstrates a great potential of the LDHF/m-mentoring approach.

Overall, the LDHF/m-mentoring arm had better skills performance at all the assessment points compared to the TRAD arm. The qualitative data also support this improvement in knowledge and skills related to maternal and newborn care and outcomes.

There was an observed drop in skills competency at three months in both arms from the immediate post-training assessment. Some of the possible explanation for the drop in skills competency is that it took time following intense training, before a practice was established as routine. Another possible cause for the drop was a health workers’ strike that was occurred just before the post-training assessments in the two states. Despite these factors, the LDHF/m-mentoring arm still performed better in all competencies at three and 12- months post-training. It is encouraging to note that this observed better performance was in competences that were very crucial for the survival of the mother and the baby on the *day of birth*; these include, AMTSL, manual removal of placenta, neonatal resuscitation, loading dose of MgSO4, and pre-eclampsia/eclampsia. The continued simulation practice may have helped in building and retaining these competencies.

Studies on BEmONC in-service training have demonstrated the value of simulation and continuous practice in improving knowledge and skills competency [7, 8]. This has led to adaptation of various approaches to determine which ones led to the greatest returns on investment [1–4, 7, 9–11]. Our findings are consistent with those of previous studies that reported that TRAD offsite training may improve the trainees’ competencies, but knowledge and skills may not be readily transferred to other co-workers at the facility, nor translated into practice or performance [13, 14]. On the other hand, methods that involve repetitive, mentor-supported learning and regularly coordinated practice sessions, such as the onsite LDHF/m-mentoring training, resulted in better skills acquisition and retention over time [1, 2, 5, 7, 8, 15–17]. This claim has been supported by other well-designed studies that showed that onsite LDHF/m-mentoring training approach, coupled with an efficient mentorship program, resulted in improved provider preparedness to deal with complications [7, 8, 16].

Findings from another study that measured patient-level outcomes showed that the LDHF/m-mentoring training approach was associated with a sustained decrease in facility-based newborn mortality and intrapartum stillbirths in Ghana [7]. The limitations acknowledged by the authors, such as the lack of a randomized control trial design and lack of concealment/information bias, have been addressed in our study using a cluster randomized controlled trial in a similar low-resource setting. Although our study did not objectively measure patient-level outcomes other than health workers report of reduced morbidities and mortalities during the qualitative study, we assessed a crucial component of the *day of birth*, namely the competency of the service providers. Our study was adequately powered to produce generalizable conclusions on the comparative effectiveness of the LDHF/m-mentoring and TRAD approaches in the study setting and possibly in similar environments especially in LMICs. As reported by other studies, to be effective, training programs should be conducted as close as possible to the workplace and be competency-based [5, 17, 18].

The present study also adds to the growing yet still insufficient body of evidence in LMICs supporting the use of simulation, specifically for training in the management of obstetrics and newborn emergencies [19–21]. Skills that are not used very frequently in management of obstetric and newborn complications are likely to be non-existent or lost due to a lack of use, and therefore the need to practice often. The appropriate dose or frequency of practice in order to reach competency level depends on many factors, which include the personal attributes of health worker among other things. Simulation-based trainings provide participants with the opportunity to repeat practice sessions in a safe environment where they will not harm patients [7, 8, 18, 21]. Using the workplace to practice skills is beneficial. The availability of the anatomical models for continuous onsite simulation practice sessions also helps to organize emergency drills to test the health systems response to life-threatening complications.

Another important finding is that some of the facilitators for the LDHF/m-mentoring approach are related to successful outcomes that were reported as health workers improved their skills, which motivated the participants to practice more and seek support from master mentors. Inadequate mentorship should be addressed to improve quality of BEmONC [22]. On the other hand, some of the barriers to this approach included lack of medical equipment and supplies, strikes by health workers, different work schedules and workplace distraction also limited peer practice sessions. To function maximally, health workers need to improve their competency and they need an enabling environment, including availability of supplies, equipment, appropriate guidelines and policies, and timely remuneration [23, 24]. Our study findings corroborated those of previous studies in LMICs on the effectiveness of the LDHF/m-mentoring training approach compared to the offsite traditional training approach to build health workers capacity [7, 8].

This study had some limitations. These include some operational-level logistic challenges in ensuring that the mentors always had the airtime in a timely manner in order to maintain seamless communication with the mentees. This was mitigated by the mentors being very understanding by using their own funds to procure air time used for m-mentoring as they awaited support provided through the study. Another limitation was the health workers’ strike which occurred around three months post-training, which negatively affected the findings at that assessment period. Finally, although the study team appealed to Ministry of Health (MoH) not to transfer the study participants to other non-study facilities for the duration of the study, some staff were transferred.

## Conclusion

This is one of the few studies from one of the LMICs that have used a pragmatic design—a cluster randomized controlled trial and qualitative methods—to generate much needed information on learning outcomes of two approaches to health worker capacity building. Our study findings corroborated those of similar studies to support the proposition to a shift from traditional offsite group-based training to low-dose, high-frequency and onsite training as an effective way to build the capacity of maternal and newborn care providers. This shift can be explored in other clinical settings to improve the quality of care. The findings suggest that a low-dose, high frequency and simulation- and practice-based training approach, including mobile mentoring is likely to result in better skills competency outcomes and higher skills retention among health workers. This study has demonstrated promising results that can address health worker absenteeism due to frequent offsite trainings, which are a major concern for health facility managers in LMICs. Training staff onsite without taking them away from service delivery points is a strong reason for adoption of the simulation-based low dose high frequency/mobile mentoring approach.

The findings are expected to provide health facility managers and other decision makers with valuable information to guide resource allocation for maximum benefit and effectiveness in building the capacity of frontline health workers. The authors recommend that capacity building among healthcare workers, to the extent possible, should utilize the onsite LDHF/m-mentoring learning approach. Further evidence is needed to determine the value of m-mentoring in influencing learning outcomes and the cost-effectiveness of both training approaches as these were not explored in our study.

## Data Availability

The datasets generated and/or analyzed during the current study are available in the figshare repository, 10.6084/m9.figshare.9955133

## Abbreviations

AMTSL: Active management of third stage of labor
BEmONC: Basic emergency obstetric and newborn care
FGDs: Focused Group Discussions
IDI: In-depth Interviews
IQR: Interquartile range
LDHF: Low-dose, high-frequency
m-mentoring: Mobile mentoring
LMICs: Low and Middle Income Countries
MCQ: Multiple Choice Questionnaire
MOH: Ministry of Health
OSCE: Objective structured clinical examination
PE/E: Pre-eclampsia/Eclampsia
PPH: Postpartum Haemorrhage
SD: Standard deviation
TRAD: Traditional group-based training
